# Conditioned soils reveal plant-selected microbial communities that impact plant drought response

**DOI:** 10.1038/s41598-021-00593-z

**Published:** 2021-10-27

**Authors:** Samantha J. Monohon, Daniel K. Manter, Jorge M. Vivanco

**Affiliations:** 1 Center for Rhizosphere Biology, Department of Horticulture and Landscape Architecture, Colorado State University, Fort Collins, CO 80523 USA; 2 USDA-ARS, Soil Management and Sugar Beet Research, Fort Collins, CO USA

**Keywords:** Microbiology, Plant sciences

## Abstract

Rhizobacterial communities can contribute to plant trait expression and performance, including plant tolerance against abiotic stresses such as drought. The conditioning of microbial communities related to disease resistance over generations has been shown to develop suppressive soils which aid in plant defense responses. Here, we applied this concept for the development of drought resistant soils. We hypothesized that soils conditioned under severe drought stress and tomato cultivation over two generations, will allow for plant selection of rhizobacterial communities that provide plants with improved drought resistant traits. Surprisingly, the plants treated with a drought-conditioned microbial inoculant showed significantly decreased plant biomass in two generations of growth. Microbial community composition was significantly different between the inoculated and control soils within each generation (i.e., microbial history effect) and for the inoculated soils between generations (i.e., conditioning effect). These findings indicate a substantial effect of conditioning soils on the abiotic stress response and microbial recruitment of tomato plants undergoing drought stress.

## Introduction

Plant-mediated rhizobacterial selection in soils is well documented^1–3^. This selective pressure is partially regulated by root exudates of plants which can alter the microbial community composition within rhizosphere soils^2^. Root exudation profiles, and subsequent microbial community assemblages, are determined by a variety of factors including genotype, plant developmental stage, exposure to various stress conditions, and plant nutrient demands^4,5^. For example, in a study performed by Santos-Medellin et al.^6^, microbial community shifts were observed under drought stressed conditions in cultivars of rice. Drought stress resulted in greater shifts in soil communities closest to plant roots, indicating strong plant influence on rhizobacteria as a result of changing environmental stimuli^6^. Although plants can modulate the microbial communities colonizing the rhizosphere, the microbial community structure is generally a combined influence of plant and native soil microbiota^7^. Therefore, decreasing the diversity and abundance of microbial communities (i.e., microbial community complexity) in soils has been shown to amplify the effects of plant selection on rhizobacterial communities^8^. A study examining the rhizobacterial communities of date palms grown in heterogeneous sites across the Sahara Desert found similar trends in microbial community composition across sites, despite differences in native soil origins^8^. These findings indicated unusually high plant influence on rhizobacterial selection, due to the decreased microbial complexity of the desert soils^8^. Other studies have looked into the artificial removal of native soil microbiota, which resulted in increased crop- or condition-specific microbial community recruitment^3,9^. Additionally, Li et al.^9^ examined the effects of autoclaving on the soil microbiome, identifying significant shifts in community composition with the presence of crops compared to soil alone conditions, indicating plant-mediated microbial recruitment. Therefore, despite generally accepted knowledge that increased microbial diversity in soils leads to benefits for plant growth^10^, decreased microbial diversity may allow for greater plant-selection of rhizobacterial membership.

Utilizing this knowledge of plant-selected rhizobacteria, researchers have investigated the ability of plant-mediated soil microbial communities to impact plant health, performance and phenotypic traits^11^. Conditioning soil communities is a way to amplify the existing microbial community for a specific plant response. For example, Panke-Buisse et al.^12^ conditioned microbial inoculants for early and late flowering in *Arabidopsis thaliana* over ten generations. Following the tenth generation, the inoculant was used on four different genotypes, all of which showed significant shifts in flowering time as a result of the inoculation treatment^12^. Furthermore, plants can condition their own soils in response to particular stress exposures. Plant modulation of rhizobacterial communities as an adaptive strategy to deal with stress conditions can be referred to as the “cry for help” hypothesis^1^, in which plants recruit the bacterial communities needed to benefit the plant under a particular stress. This phenomenon has been observed for biotic and abiotic stresses, including soil communities aiding in pathogen and herbivory resistance^1,13^.

Suppressive soils are a well-known example of the effects of conditioned microbial communities on plant health and performance under biotic stressors^14^. These adaptive soils have two generally accepted types of suppression: specific and general^1,14,15^. Within both types of suppression, a microbially-mediated plant defense against soil pathogens is induced by the recruitment of particular beneficial microbes. General suppression is a whole community response, in which, native rhizobacteria outcompete the pathogen for available resources creating low levels of protection against a variety of different pathogens^15^. Specific suppression, however, is due to key players in the microbial community that aid in protecting the plant from infection through directly damaging the pathogen or indirectly inducing plant defense responses^14–16^. This type of specific suppression has occurred in soils of wheat and barley monocultures. A phenomenon called Take-all decline (TAD), is a conditioned response of soil communities to continued exposure to Take-all disease, caused by *Gaeumannomyces graminis* var. *tritici.* Plants infected with *G. graminis* var. *tritici* have shown a consistent trend of plant infection for several years followed by sudden plant resistance after continued monocropping of wheat and barley^14–16^. These findings show plants’ abilities to select for beneficial microbial communities as a successful stress defense strategy.

Suppressive soils have led to many other recent studies looking into the conditioning of soil microbial communities as an adaptive strategy for crop resilience to other biotic and abiotic stresses^2,13,17^. Here, we used the combined understanding of suppressive soils, transferrable microbial inoculants, and artificially lowering the complexity of soils (by means of autoclaving), to investigate plant-mediated conditioning of drought-resistant soils. We reasoned that through generational conditioning and amplified plant influence of drought-specific microbial communities, we could reveal plant-selected microbial taxa which benefit plant health and performance under severe drought conditions.

## Results

### Autoclaved soil studies

#### Plant DW biomass differences as a result of inoculation with historically conditioned microbes

**Figure 1:**
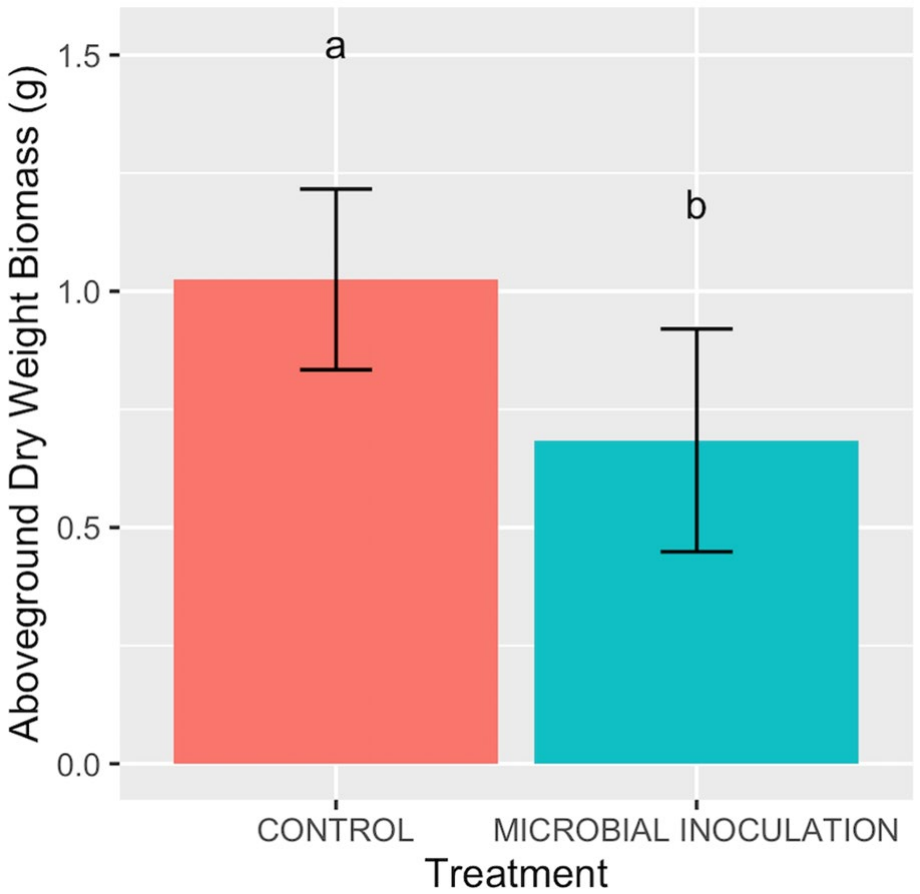
Total plant DW biomass of tomatoes under control and microbial inoculation treatments. Values indicate results from the combined generations. Red bars represent control treatments with no inoculation. Blue bars represent inoculated treatments. Different letters indicate significant differences at p < 0.05.

Plant dry weight measurements (DW) were taken for below and above-ground biomass. The results showed no significant difference in DW biomass as a result of conditioning effects (p > 0.05). A one-way ANOVA was performed to analyze the difference in plant DW biomass between the combined treatment groups from both generations (Fig. 1). Plants given an inoculation showed significantly decreased DW biomass as compared to control plants (p < 0.001) with a 33.23% percent decrease in total DW biomass.

A one-way ANOVA was performed to analyze differences in above- and below-ground plant DW biomass between treatment groups. Below-ground DW biomass showed a slight significant decrease as a result of microbial history (p = 0.027). Above-ground plant DW biomass showed a significant decrease as an effect of microbial history (p < 0.001). Below-ground and above-ground results showed no significant effect of conditioning.

It is important to note that beyond size, there were no visual differences between plants given inoculations as compared to those in the control treatments for either generation.

### Effects of inoculation treatment on alpha diversity of microbial communities

Alpha diversity was calculated at the ASV level using the Shannon Diversity index and the observed richness values. A two-way ANOVA was performed using the values calculated from both indexes. The results showed no significant difference in alpha diversity between inoculated and control treatments, for either measure of alpha diversity (Figures S1, S2). These results indicate that, despite shifts at the whole community and taxa level for microbial community structure and abundances as a result of inoculation, the alpha diversity values were not affected.

### Effects of microbial history on rhizobacterial communities

#### Microbial community differentiation as an effect of microbial history

**Figure 2:**
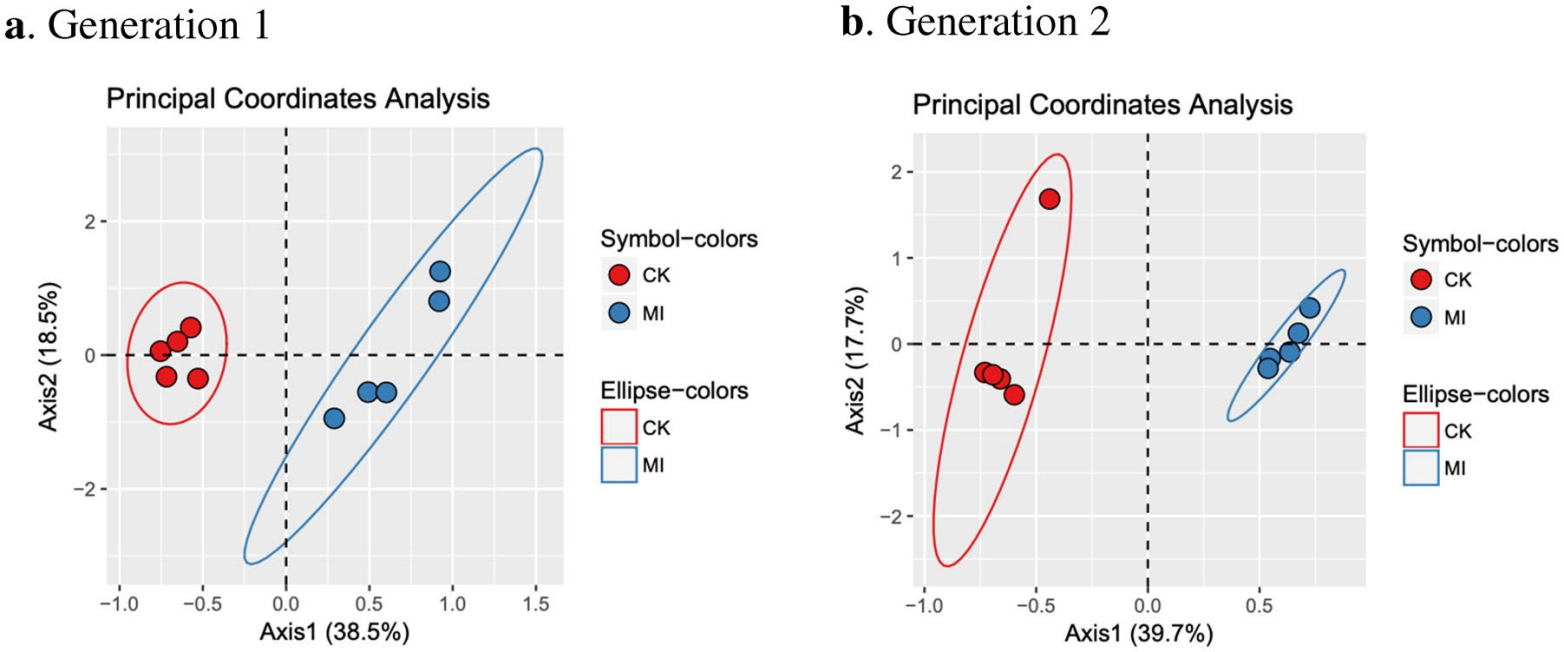
Principal coordinates analysis (PCoA), using Bray–Curtis distances, representing rhizobacterial communities of soil samples from control and microbial inoculation treatments (n = 5 soil samples per water treatment) within generation 1 (**a**) and generation 2 (**b**). Red and blue circles represent control (CK) and microbial inoculation (MI) treatments, respectively.

Two principal coordinates analyses (PCoA) were performed to visually represent differences between inoculation and control treatments groups within each generation. Analyzing differences between treatment groups within a given generation revealed the effects of microbial history on the resulting microbial communities. We observed a shift in microbial community structure with inoculation as compared to control treatments for both generation 1 (Fig. 2a) and generation 2 (Fig. 2b). A perMANOVA, using Bray–Curtis distance matrices at the ASV level, revealed a significant difference between microbial communities when comparing the inoculated and control groups within generation 1 (p = 0.005) and generation 2 (p = 0.005). These results indicate a significant impact of microbial history on resulting rhizobacterial communities.

#### Mimicking microbial history of soils affects genus-level microbial community abundances

Differential abundance analyses were performed to determine taxa, to the genus-level, that significantly differed as a result of microbial inoculation. Genera that showed significantly different abundances in inoculated compared to control soils were identified for generation 1 (Table S1) and generation 2 (Table S2). The fluctuations in abundance of these taxa can be attributed to changes in the microbial history of the rhizosphere soils. Among these differing taxa, *Xanthomonadaceae_unclassified* showed a significant increase in inoculated treatment groups within both generations, indicating potential plant selection for this bacterium under severe drought conditions. Additionally, *Bryobacteraceae_unclassified* and *Comamonadaceae_Hydrogenophaga* showed significantly increased abundances as an effect of microbial inoculation in generation 1. *Phyllobacteriaceae_Chelativorans, Phyllobacteriaceae_Aminobacter* and *Oxalobacteraceae_Janthinobacterium* showed a similar significant increase in abundance as an effect of microbial inoculation in generation 2. Alternatively, *Chthoniobacteraceae_unclassified* showed a significant decrease in inoculated soils as compared to control soils within each generation. These results show that this taxon is consistently restricted in abundance when given an inoculant with microbes historically conditioned for severe drought stress, suggesting that this bacterium is not selected or actively restricted by plants under drought stress.

### Effects of conditioning on rhizobacterial communities

#### Microbial community differentiation of microbiomes conditioned under severe drought stress over generations

**Figure 3:**
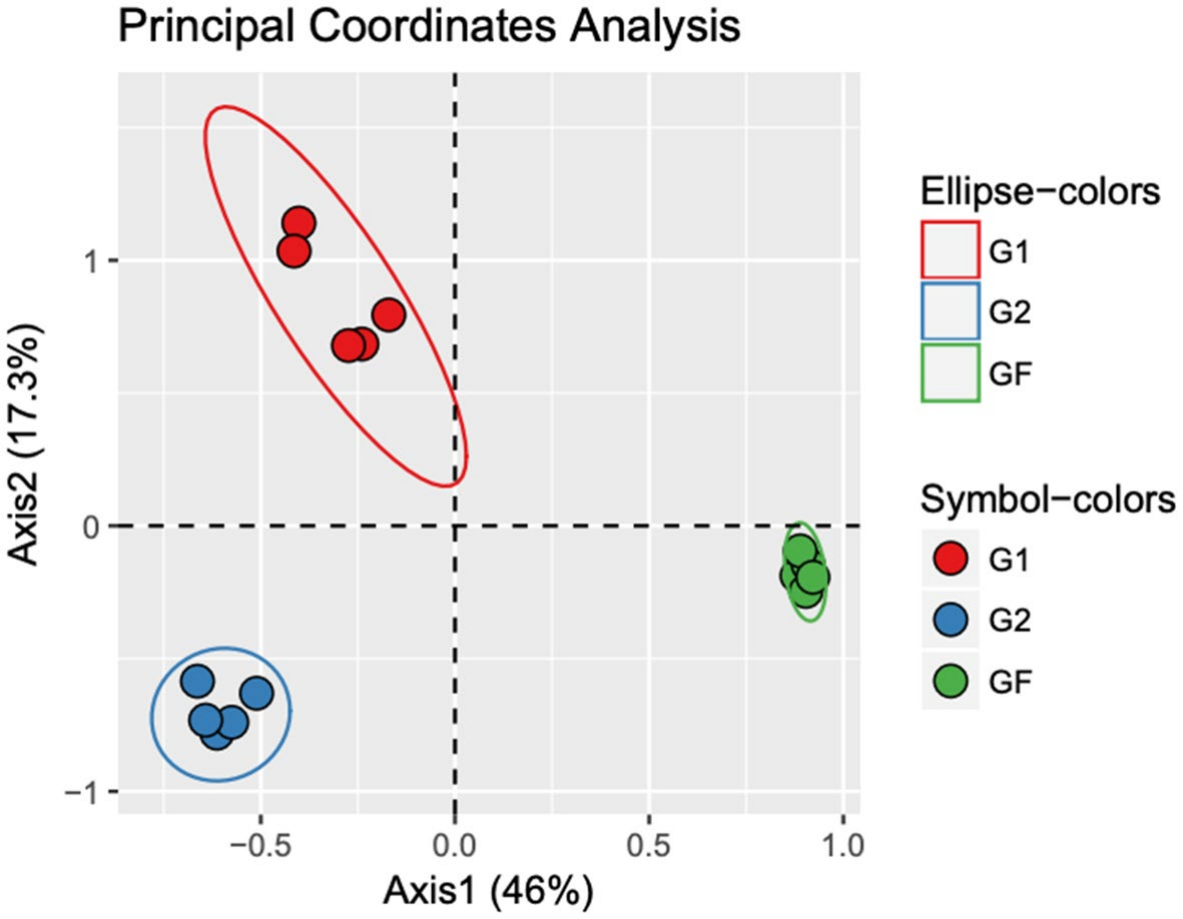
Principal coordinates analysis (PCoA), using Bray–Curtis distances, representing rhizobacterial communities of soil samples from inoculated treatments (n = 5 soil samples per water treatment) from generation 1 (G1), generation 2 (G2) and the initial soil slurry (GF). Green, red and blue circles represent GF, G1 and G2, respectively.

A principal coordinates analysis (PCoA) was performed to visually compare microbial communities from each generation of severe drought conditioning, including the microbial composition from the initial soil slurry community (i.e., generational foundation [GF]) (Fig. 3). This analysis identified the impact of conditioning soil communities over generations under a given condition and specific crop-type. The PCoA revealed significantly different microbial communities from soil samples resulting from each generation. A permutational analysis of variance (perMANOVA), using Bray–Curtis distance matrices at the ASV level, was used to verify significant differences visually observed. These data showed significant community shifts of rhizobacterial communities as an effect of generation (p = 0.001). These findings suggest an impact of conditioning soils on microbial community composition with each additional planting.

#### Effects of severe drought conditioning on genus-level microbial community abundances

Differential abundance analyses were performed to determine taxa, to the genus-level, that significantly differed in abundance between treatment groups. To best identify key players in the conditioning of soil microbial communities under severe drought stress over generations, comparisons were made between inoculation treatments from the initial soil slurry, generation 1 and generation 2. In this way, we were able to identify genera that increased or decreased in abundance with continued exposure to severe drought conditions. Genera which significantly differed in abundance between inoculated soils from generation 1 and generation 2 were identified (Table S3). Additionally, taxa observed to significantly change in abundance from the initial soil slurry as compared to the inoculated soils from generation 1 or generation 2 were recorded (Tables S4, S5). Genera which showed increasing trends with generational conditioning of soils were identified. These taxa showed significant increases in abundances for at least two of the previously mentioned comparisons between inoculation groups from differing generations (Table 1; Tables S3–S5). Additionally, all of these taxa were observed to show significant increases in abundance in generation 2 inoculated treatment as compared to the initial soil slurry microbiome. The shifts in abundance of these taxa suggest that these genera were selected by tomato plants under severe drought conditions over continued exposure.

**Table 1:**
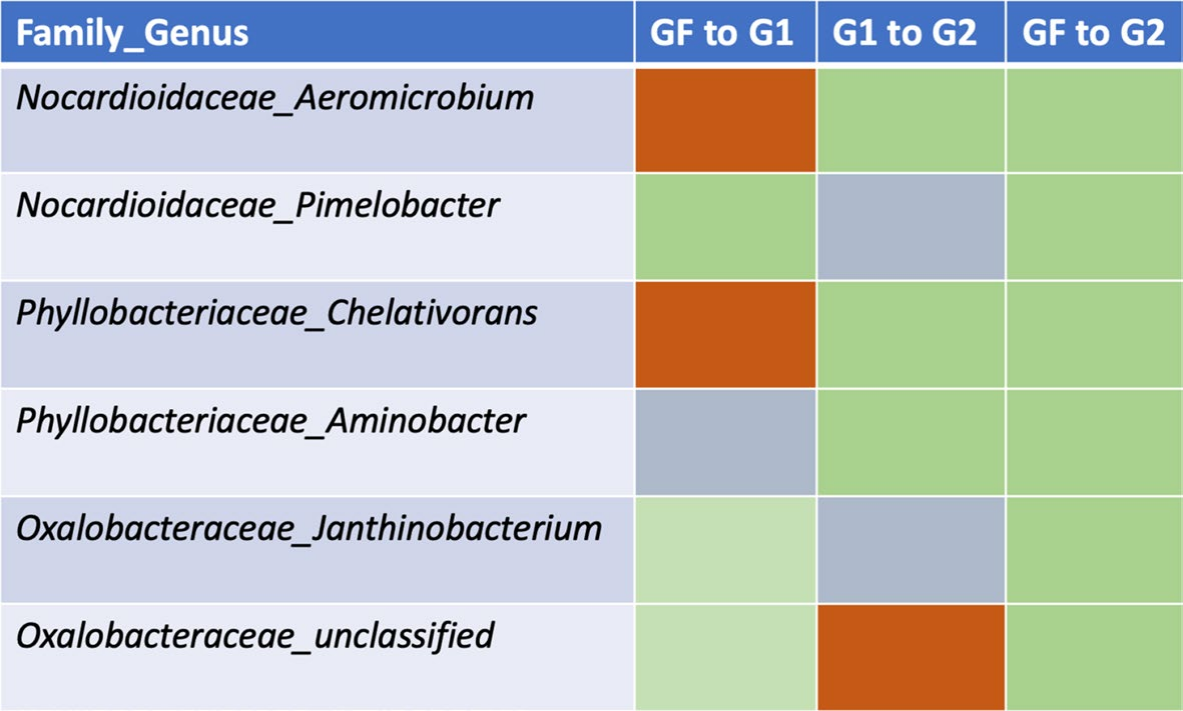
Bacterial taxa, to the genus level, which showed differing abundances when comparing inoculated treatment groups from different generations.

GF signifies microbial community membership from the initial soil slurry used to inoculate the first generation. G1 and G2 represent generation 1 and generation 2, respectively. Green boxes represent a significant increase in abundance and red boxes represent a significant decrease in abundance. Gray boxes represent no significant differences in abundance between the two treatment groups. The two generational treatments used in a comparison are identified at the top of each column.

### Not autoclaved soil studies

#### Comparative effects of inoculation on plant performance in autoclaved and not autoclaved soils

Plant DW biomass measurements were taken and total DW biomass was compared between treatment groups within autoclaved and not autoclaved soil conditions. We performed a two-way ANOVA to compare the differences in DW biomass for plants with inoculation under autoclaved and not-autoclaved conditions within each generation. These comparisons provided insight into the inoculation’s ability to impact plant performance under varying degrees of contemporary microbial complexity. As previously mentioned, inoculation treatments for autoclaved soils in both generations showed a significant decrease in plant biomass (Fig. 1). In not autoclaved soils, there was no significant effect of inoculation treatment on plant DW biomass (p > 0.05).

#### Conditioning effects of microbial community composition within not autoclaved soils

Soils that were not autoclaved showed no significant shifts in microbial community composition as a result of conditioning. A principal coordinates analysis (PCoA) was performed to visually compare microbial communities from inoculated and control soil treatments within each generation. This allowed us to observe differences in rhizobacterial communities as a result of inoculation of previously conditioned microbes, within a high complexity soil environment. A permutational analysis of variance (perMANOVA), using Bray–Curtis distance matrices at the ASV level, showed no significant differences between inoculated and not inoculated treatments for either generation. However, there was a visual increase in microbiome differentiation between generation 2 treatment groups as compared to the overlapping microbial communities from generation 1 treatment groups (Figure S3).

#### Effects of conditioning and microbial history on genus-level microbial community abundances

We analyzed the bacterial abundances of two taxa of interest within not autoclaved soil treatments, *Phyllobacteriaceae_Aminobacter* and *Phyllobacteriaceae_Chelativorans*, to determine differences in rhizobacterial recruitment as a result of higher soil microbial complexity. We performed differential abundance analyses to identify abundance changes as an effect of microbial history and soil conditioning. In contrast to autoclaved soil conditions, there were no significant differences in abundance observed for either bacteria within not autoclaved soils. However, there were slight differences in abundance when comparing different treatment groups within and between generations (Tables S6, S7). These findings suggest the interference of native soil microbiota in the selection of these soil taxa.

## Discussion

### Plant selected microbial communities aid in drought avoidance strategy

We found that tomato plants given microbial inoculants conditioned for monocultured tomato plants under severe drought stress, showed significantly decreased plant DW biomass as an effect of microbial history (Fig. 1). Furthermore, when broken down by above- and below-ground biomass measurements, statistical analyses revealed greater differences in above-ground biomass as an effect of microbial history. Therefore, plants with microbial communities previously exposed to severe drought stress showed slower vegetative growth than those without inoculants. Reduced size was the only visual effect of the inoculation treatment; there were no signs of pathogenic attack, nutrient deficiency or other stress response in the plants. Our findings indicate a potential adaptation in the functional traits of the microbial communities within the inoculation treatment, as a result of prior exposure to severe drought stress. This reduction in growth rate, in combination with a lack of other visual plant stress responses, may be an alternative adaptive strategy for plants to increase drought resilience.

There are three known strategies plants use in dealing with drought stress: drought tolerance, drought escape and drought avoidance^18–20^. Drought tolerance is manifested by a plant’s continued normal growth and function, despite drought exposure^18^. Drought escape is a tactic used by plants in which drought stress triggers rapid development to reach reproductive stages before the onset of harsher conditions^20^. Drought avoidance, sometimes called phenotypic flexibility, is a strategy in which plants change their morphology or physiology in order to maintain water status in certain organs. This strategy can result in a wide range of altered plant traits including changes to stomatal rates or abundance, slowed plant growth, increased root size, or decreased leaf area and size^21^. The decreased plant biomass we observed, as a result of microbial inoculation, suggests that the microbial community may be employing a drought avoidance strategy to better help the host plant deal with the severe drought stress. Additionally, the root biomass was unaffected by inoculation, indicating that the vegetative growth, the area of the plant responsible for stomatal opening and transpiration rates, was the only plant compartment affected. Thereby, further indicating a drought avoidance strategy utilized to maintain normal water status under severe drought stress. A meta-analysis performed by Li et al.^22^, showed that in contrast to popular breeding strategies for drought tolerant traits in wheat cultivars, wild cultivars with greater drought avoidance strategies actually showed greater overall yields and resulting aboveground biomass under severe drought conditions. However, the domesticated cultivars bred for drought tolerant traits showed greater success under less severe drought stressed conditions^22^. This study supports our findings that drought avoidance strategies may be beneficial to plants under more severe drought conditions.

Additionally, in a study performed by Bresson et al.^23^, a PGPR strain, *Phyllobacterium brassicacearum* STM196, was inoculated into soil communities of *Arabidopsis* plants. Plants exposed to water deficit conditions and given this inoculation were shown to have delayed growth rates compared to plants under water deficit without the inoculation. Interestingly, the plants with the PGPR inoculant showed greater plant biomass at the time of bolting, despite reduced vegetative growth leading up to bolting stage. These findings suggest that *P. brassicacearum* STM196 restricted the plant’s rate of development and subsequently, prolonged vegetative growth, allowing the plant to accumulate greater biomass over a longer period of time^23^. Similar to these findings, we observed decreased plant growth as a result of microbial inoculation under severe drought stress. Additionally, our microbial analysis identified *Phyllobacteriaceae_Aminobacter* and *Phyllobacteriaceae_Chelativorans*, two other members of the *Phyllobacteriaceae* family, as increasing with soil conditioning and microbial inoculation. These two taxa showed significantly increased abundances under inoculated treatment as compared to control soils in generation 2 (Table S2). Abundances also increased with conditioning from generation 1 to generation 2 as well as overall conditioning effect from the initial soil slurry to generation 2 (Tables S3–S5). Further research is needed to understand the effects of these specific taxa on tomato plant morphology and the final biomass measurements at flowering or yield, however, our results indicate that these taxa may be key drivers in the reduced plant biomass accumulation as a result of inoculation.

### Soil conditioning affects plant performance and rhizobacterial community composition

Conditioning soils for tomato planting under severe drought treatment led to significant effects on microbial community assemblage. We observed significant microbial community shifts following each generation of conditioning, beginning with the initial soil slurry inoculant (Fig. 3). These effects were seen on the whole community level and in specific bacterial abundances. These shifts were expected, as soils exposed to consistent plant types and environmental conditions over generations have been shown to alter microbial communities of plants^14^. Furthermore, we observed changes in plant DW biomass as a result of microbial inoculations (Fig. 1). As mentioned above, we suggest that the observed decrease in biomass may be indicative of increased drought resilience by reducing growth rates of host plants. The changes in microbial community membership and plant phenotype are reminiscent of studies observing suppressive soils. In these studies, monocultured crops under consistent biotic stress are able to alter microbial community composition in a way that eventually results in resistance to pathogenic attack^1,14,15^. Researchers have begun to investigate similar conditioned responses of soils for drought resistance, including a study performed by Lau and Lennon in which phenotypic changes in plants grown in soils conditioned under drought stress were observed^13,17^. This study, in combination with more recent findings indicating prior drought exposure as a benefit for plant resistance to contemporary drought conditions^24^, suggests that conditioned soils have the potential to develop resistance against drought. The effects of conditioning observed in our study further these findings by identifying specific taxa related to these microbial and phenotypic changes.

### Genus level impacts of conditioning and microbial history lead to drought-specific microbiome

Genera that showed significant differences in bacterial abundances as a result of conditioning and microbial history effects were identified to determine key taxa associated with the drought stress responses observed. Conditioning effects are seen when comparing microbial communities over generations of soils exposed to severe drought and tomato plantings; microbial history effects are those differences observed within a given generation as a result of soil inoculant. Notable taxa, to the genus level, that were significantly impacted by microbial history were *(O: Gemmatimonadales) unclassified*, *Bryobacteraceae_unclassified*, *Chthoniobacteraceae_unclassified* and *Xanthomonadaceae_unclassified. (O: Gemmatimonadales) unclassified* and *Chthoniobacteraceae_unclassified* both showed a trend towards decreased bacterial abundance with inoculation of microbes historically conditioned under severe drought stress (Tables S1, S2). *Chthoniobacteraceae_unclassified* showed significant declines in abundance when comparing inoculated and control soils for both generations. We observed a decrease in abundance of *(O: Gemmatimonadales)* with inoculation for generation 1 only. These findings suggest that these taxa may be susceptible to drought conditions and not needed for plant survival under drought stress. In contrast, *Bryobacteraceae_unclassified* and *Xanthomonadaceae_unclassified* showed significant increases as a result of inoculant with historically conditioned microbial communities for both generations. *Phyllobacteriaceae_Chelativorans, Phyllobacteriaceae_Aminobacter* and *Oxalobacteraceae_Janthinobacterium* showed a similar significant increase in abundance as an effect of microbial inoculation in generation 2. These data suggest that these bacteria, showing increased abundances as an effect of microbial history, may have been selected for under drought stress conditions in the prior generations. Additionally, these bacteria may aid in the size restriction we observed in inoculated treatment groups.

Conditioning effects influenced abundance levels of a few notable taxonomic groups, including *Nocardioidaceae_Aeromicrobium, Nocardioidaceae_Aeromicrobium, Phyllobacteriaceae_Chelativorans Phyllobacteriaceae_Aminobacter, Oxalobacteraceae_Janthinobacterium* and *Oxalobacteraceae_unclassified.* These genera all showed trends of increased abundance levels as a result of conditioning soil communities under tomato cultivation and severe drought conditions. This was quantified using differential abundance analyses comparing genus-level abundances in inoculated treatment groups from generation 1, generation 2 and the initial soil slurry microbial community (Tables S3–S5). All of these bacteria showed an increase in at least 2 generational comparisons including an overall increase as an effect of conditioning when comparing abundances in the initial soil slurry to generation 2. Members of the *Oxalobacteraceae* family are known to have drought tolerant traits, including ACC deaminase production^25^. Furthermore, members of this family have been found in soil rhizobacterial communities native to arid regions and have been shown to be successful inoculants to induce drought tolerance in susceptible crops^26,27^. Additionally, relative of the *Nocardioidaceae* family, have been shown to be important microbial community members in drought-related soil studies. For example, *Nocardioides albus EN46*, was identified as benefitting Arabidopsis plants under biotic stress by promoting defensive priming of stress response pathways^28^. Furthermore, the order, *Actinomycetales*, and phyla, *Actinobacteria,* have been identified as important rhizobacterial members influencing plant performance and drought tolerance in other studies^29,30^. The genera *Phyllobacteriaceae_Chelativorans* and *Phyllobacteriaceae_Aminobacter* were observed to increase in abundance as a result of both conditioning over generations and microbial history within generation 2 (Tables S1–S5). This taxon is discussed previously and may be a key driver of the plant size restriction we observed as a result of inoculation.

Specific suppression in pathogen-resistant soil systems is due to key players in the microbial community that directly or indirectly provide defense for host plants^14–16^. For example, recent findings have shown that the rhizobacteria responsible for the suppression of Take-all disease in barley and wheat monocultured systems is 2,4-diacetylphloroglucinol (2,4-DAPG)-producing *Pseudomonas* *fluorescens*^14,31^. Similar to these findings, our key taxa identified here as having increased in conditioned soils, may aid in the drought stress resistance of tomatoes under continued drought stress. Here we have identified these taxa in the conditioning of drought exposed soils, however, future research is needed to determine the direct and indirect effects of these bacteria on tomato plants and microbial community composition.

### Higher microbial community complexity weakens conditioning effects

Suppressive soils often take several years in field conditions to create resilient responses in plants, therefore, by lessening microbial complexity we hoped to accelerate this conditioning effect in a greenhouse experiment. Our results revealed that inoculation had a significant effect on plant DW biomass in autoclaved soils (Fig. 1), however, under not autoclaved soil conditions, we found no significant difference in biomass as an effect of inoculation (p > 0.05). Native microbial complexity is known to have an influence on rhizosphere communities^7^. Furthermore, it has been documented that lower soil microbial complexity allows for greater plant influence as compared to soils with complex communities of native soil microbiota^8^. For this reason, we expected to see a weakened impact of both plant selection and microbial inoculant effects on contemporary plant performance and microbial community composition. This furthers the importance of utilizing lower microbial complexity when working in a controlled, greenhouse setting to determine plant influence on microbial recruitment. Additionally, microbial community composition in generation 1 as compared to generation 2 showed greater differentiation between inoculated and control treatments, although not statistically significant (Figure S3). This suggests that there may be a delay in the effects of soil conditioning and microbial inoculation under soils with higher microbial complexity.

Similar weakened effects were seen at the genus-level. In not autoclaved soils, *Phyllobacteriaceae_Chelativorans* and *Phyllobacteriaceae_Aminobacter* showed no significant differences in abundances when analyzing the effects of conditioning or microbial history (Tables S6, S7). However, *Phyllobacteriaceae_Aminobacter* showed a similar pattern to the differences in abundance seen in autoclaved soils, with a slight increase in generation 2 inoculated treatment as compared to control and a slight increase in abundance in generation 2 as compared to generation 1 inoculated treatments. *Phyllobacteriaceae_Chelativorans* showed no differences in abundance when analyzing microbial history or conditioning effects. Furthermore, these taxa showed slight decreases when comparing the microbiome of the initial soil slurry to that of the inoculated treatment in generation 2. These results indicate that the higher microbial complexity of not autoclaved soils interfered with the selection of these taxa under severe drought conditions. This is a similar pattern seen in suppressive soils when a different crop, for example alfalfa or oat cover crop, is planted in a monocultured system and disrupts the conditioned soil microbiome^32^. In these soils, the pathogen resistance is negatively impacted by the interference of a new crop and set of root exudates, similar to the disruption we saw in our soils with the addition of greater amounts of native soil microbiota.

## Conclusions

Our findings suggest significant impacts of soil conditioning and microbial history on plant performance and microbial community composition for tomato cultivation under severe drought stress. Microbial inoculation of soils previously conditioned for similar stresses and crop type have been shown to increase plant resistance to abiotic and biotic stressors. Our results suggest a similar trend through the microbially-mediated restriction of plant vegetative growth under drought stress. This strategy can be a way for plants to better regulate water loss under severe drought conditions. Furthermore, we identified a lack of significant impact of soil conditioning and microbial history on soils with higher microbial complexity. Thereby, indicating the importance of amplifying plant influence on soil microbial communities to accelerate the effects of soil conditioning for greenhouse experiments. Further research is needed to understand impacts of conditioned soil communities on resulting biomass and yield outcomes.

## Methods

### Collection and autoclaving of soils

Soil was collected in June of 2020, from a USDA-certified organic cover crop field (Agricultural Research, Development and Education Center [ARDEC] South, Specialty Crops program, Fort Collins, CO), most recently having grown peppers and melons. Bulk soil was collected as well as rhizosphere soils shaken from the roots of melon and pepper plants. Soils were sifted through a No. 10 metal sieve (2 mm wide). Following sieving, half of the soil was exposed to steam sterilization using a STERIS brand autoclave for three 40-min liquid cycles at 121 °C and is referred to as autoclaved soils. The remaining soil was not autoclaved and is referred to as not autoclaved soils. Both autoclaved and not autoclaved soils were dried out in the greenhouse prior to weighing and filling pots.

Autoclaving of soils was used to lower initial microbial diversity and abundances of soil microbial communities (i.e., microbial community complexity). This allowed us to observe the effects of decreased microbial competition, and subsequent increased plant influence, on rhizobacterial recruitment. Amplifying plant-selection of microbial communities was used to promote rapid adaptations of soil communities for tomato growth under severe drought stress.

Prior studies utilizing autoclaving as a tool for soil sterilization have shown slight shifts in nutrient content following autoclaving^9^. A nutrient analysis was performed on the autoclaved and not autoclaved soils in our study to determine any differences in NPK content (data not shown). The analysis was performed by WARD Laboratires, Inc. The results for the control soils showed total Nitrate–N, available K and available P were 5.9 ppm, 452 ppm and 94 ppm, respectively. The total Nitrate–N, available K and available P for autoclaved soils were 5.8 ppm, 413 ppm and 86 ppm, respectively. These results indicate no notable shifts in nutrient content that may further affect results from our study.

### Experimental design

Burpee Rutgers variety tomato seeds (*Solanum* *lycopersicum* L.) were surface sterilized with 3.0% NaClO, rinsed three times with sterile water, and imbibed in sterile water for 24 h prior to planting. Seeds were planted in sterile full-strength MS media in petri dishes. Seeds were placed in a growth chamber for 11 days, allowing for germination and root and shoot emergence. After 11 days, seeds were transplanted into plastic pots filled with 350 g of dry autoclaved or not autoclaved soils. For each soil treatment, plants were grown under severe drought (SD) conditions at 55% Field Capacity for 3 weeks. This was a two-generation study in order to determine the potential impacts of a conditioned, drought stress-specific microbiome on plant performance under severe drought stress.

Each generation had four treatments groups, including an inoculated and control treatment for both autoclaved and not autoclaved soil (with seven pots per treatment: total n = 28). Soil slurries for inoculants were derived from a previous study with tomato seedlings grown under severe drought conditions in autoclaved soils^33^. The second generation had inoculants made from autoclaved and not autoclaved soils from the preceding generation. Rhizosphere soils (23.33 g) from the top three performers in the inoculation treatment from generation 1 were pooled to create a total of 70 g of rhizosphere soil. Performance was determined by plant fresh weight biomass, root:shoot ratio and height. Slurries were created using the method from Panke-Buisse et al.^12^ and inoculated 3 days after transplanting for both generations.

Inoculation treatments and controls were used to determine the efficacy of utilizing microbial communities from plants previously exposed to severe drought stress as a tool to infer greater drought resilience to host plants. These effects, referred to here as microbial history effects, were measured by comparing plant performance and resulting microbial communities of inoculated and control conditions under severe drought conditions. The generational design of this study was used to uncover specialized microbial functions which induce drought resilient traits for host plants as a result of continued exposure to severe drought stress. These effects, referred to here as conditioning effects, were analyzed by identifying trends in plant performance and microbiome composition between generations for inoculated treatment groups. There was no absolute control used in this study, in the form of a well-watered treatment group, because the effects of drought stress on tomato plants are already known. The focus here was the effects of conditioning and microbial history on similarly drought stressed crops.

### Drought conditions

Seedlings were watered regularly for 4 days following transplanting to allow plants to successfully establish prior to drought stress. Induction of drought was 5 days after transplanting. Plants were grown under severe drought conditions based on the field capacity of the soil. Percent moisture was determined using the moisture tension method at Colorado State University’s Soil, Water and Plant Testing Laboratory**.** The field capacity percentages were calculated based on weight, using 55% for severe drought (SD) conditions. Three random pots from each treatment were weighed daily between 2:00 and 3:00 p.m. The average weight was used to determine the amount of water needed to maintain the 55% field capacity. The weight of the soil and pot were known and included in calculations to replace the water lost by transpiration and evaporation. At week 2, plants were lined up by size within each treatment and a replicate visually closest to the average size for each treatment was chosen. The chosen plants were harvested and the fresh weight above- and below-ground biomass was measured. This number was used in future measurements to compensate for the weight of the plant in field capacity calculations. The experiment was conducted at CSU’s Horticulture Center Greenhouse Facility.

### Plant data collection

Drought was induced for 3 weeks for each generation, after which plants were harvested. Relative Water Content (RWC) was measured according to Yuan et al.^34^. Three randomly selected leaves from each plant were submerged in Milli-Q water for 24 h. Relative water content (RWC) was calculated in plants as follows: (Fresh weight − Dry weight)/(Turgid weight − Dry weight) × 100^35^. On the same day, height was measured, plants were cut at the root-shoot axis and fresh-weight measurements were taken. Rhizosphere soils were collected for each plant by gently shaking soils off roots and storing in Ziploc bags. Above- and below-ground plant parts were placed in paper bags and dried in an oven for 72 h at 65 °C. Dry-weight measurements were taken following drying. Above- and below-ground dry-weight biomass measurements were used as an indicator for drought stress effects on tomato plants. This is a common practice used in drought stress studies for tomatoes^36,37^ and other plant types^38–41^.

We identified differences in total DW biomass between treatment groups as an effect of microbial history or conditioning. Inoculation treatments were compared under autoclaved soil conditions within each generation to determine the microbial history effects on plant performance; significant differences were analyzed for inoculation treatments between generations to determine the effects of conditioning on plant performance. Generation 1 inoculations were created from soil slurries containing microbial communities conditioned for tomato growth under severe drought stress, thereby, mimicking the plants’ contemporary conditions.

A one-way and two-way ANOVA were performed using R Studio (Version 1.2.5033) to analyze plant height, RWC, DW biomass, root length and root area in both generations.

### Soil DNA extraction

Genomic DNA (gDNA) was isolated from rhizosphere soil samples. Five rhizosphere soils were used for five total gDNA samples per treatment. Samples were extracted using Qiagen DNeasy PowerSoil Kits, according to the manufacturer's instructions. Nucleic acid concentration and sample purity were quantified and determined via the use of a NanoDrop 2000 Spectrophotometer (Thermofischer). DNA samples were then stored at − 80 °C prior to Illumina MiSeq library preparation and downstream microbiome analyses.

### Library preparation for illumina MiSeq sequencing

Initial soil gDNA samples were diluted 1:20 with molecular water to reduce PCR inhibitors introduced during DNA extraction. Quantitative PCR targeting the V3–V4 region of the bacterial 16S rRNA gene was performed using a modified version of primer set 341F/785R used in Li et al.^9^. This qPCR reaction was performed in 20 μL reaction volumes containing 2 μL of template DNA and 18 μL of the master mix. The master mix consisted of 10 μL 2× Maxima SYBR Green (Thermo Scientific, Waltham, MA, USA), and 2 μL each (10 μM) of forward and reverse primers and brought to a total volume of 18 μL using 4 μL of molecular grade water. The PCR thermal cycling conditions were as follows: 95 °C for 5 min, 35 amplification cycles (94 °C for 15 s, 55 °C for 30 s, 72 °C for 60 s) followed by a final annealing stage at 72 °C for 5 min to reduce chimeric reads. A standard curve using purified *Psuedomonas putida* KT2440 gDNA was run with the samples to quantify the starting rRNA copies per g^−1^ soil. Resulting amplicons were then purified using an in-house preparation of solid phase reversible immobilization (SPRI) magnetic beads^9^.

A second PCR cycle was then conducted to attach unique Illumina Nextera XT indices to each bead cleaned sample for subsequent sample demultiplexing. Each well contained 5 μL of first round and bead-cleaned qPCR product, 25 μL of 2X Maxima SYBR Green (Thermo Scientific, Waltham, MA, USA), 5 μL each of both forward and reverse indices were combined along with 10 μL of water, bringing the total volume to 50 μL. PCR conditions were as follows: 95 °C for 3 min, eight amplification cycles (95 °C for 30 s, 55 °C for 30 s and 72 °C for 30 s) followed by final annealing of 72 °C hold for 5 min. The resulting PCR product was again SPRI-bead cleaned using the same methods previously mentioned. Amplicons were then quantified using a Qubit fluorometer (Thermo Scientific, Waltham, MA, USA) prior to normalization and pooling. The final pool was run on a TapeStation system (Agilent Technologies, Santa Clara, CA, USA) to determine size and purity of amplicons, and Kapa Biosystems (Sigma-Aldrich, St Louis, MO, USA) qPCR was performed according to the manufacturers’ instructions to determine concentration. The final pooled sample was diluted to 4 nM and the DNA library was denatured with 0.2 N NaOH, diluted to 10 pM using provided HT1 buffer, and spiked with 20% PhiX library standard diversity-control. Illumina’s MiSeq v3 600-cycle Reagent Kit (Illumina, San Diego, USA) was used for library dilution and loading onto the MiSeq at CSU’s Next Generation Sequencing Laboratory (Fort Collins, CO).

### Bacterial 16S rRNA gene sequence analysis

De-multiplexed raw fastq files were processed with the DADA2 pipeline using R Studio’s Bioconductor packages^42^. Briefly, all primers were removed from each sequence using the open source Python program Cutadapt^43^ and amplicon sequence variants were inferred using the default pipeline in DADA2. Each sequence variant identified in DADA2 was classified to the closest reference sequence contained within the Green Genes 13_5_99 reference database with at least a 99% sequence similarity using Vsearch global aligner^44^. Each taxonomic profile assigned was used to determine bacterial genus and species-level relative abundance values. Classified sequences were used to generate collapsed abundance tables at the genus and species level. Downstream analyses were conducted using R Studio’s phyloseq package^45^ or myPhyloDB (v 1.2.0)^46^. Samples were rarified at a cutoff of 5000 reads using myphyloDB prior to downstream analysis applications using myphyloDB or R Studio. All samples met rarefaction criteria and no samples were removed from downstream analyses. Measurements of α-diversity assigned to treatments were determined using the Shannon diversity index and observed richness. A two-way ANOVA was used to compare mean α-diversity values of inoculated and control treatment groups in autoclaved soils. Values from the Bray–Curtis dissimilarity index were calculated in myPhyloDB from the ASV abundance tables and used to quantify differences in microbial community structure between samples from different treatments and generations. edgeR was used to analyse differential expression using the negative binomial distribution^47^. The myPhyloDB software was then used to visually represent distances using principal coordinates analyses (PCoA). A complementary non-parametric multivariate statistical test, a permutational analysis of variance (perMANOVA), and differential abundance analyses (FDR < 0.1) were performed to determine differences in microbial communities between treatments^46^.

## Supplementary Information

Supplementary Figures.

Supplementary Tables.

## Data Availability

The data that support the findings of this study will be available in myPhyloDB at https://myphylodb.azurecloudgov.us/myPhyloDB/home/ after publication. This study complies with local and national guidelines.
