# Supplementary material for: Conditioned soils reveal plant-selected microbial communities that impact plant drought response
Source: Sci Rep. 2021 Oct 27;11:21153. doi: 10.1038/s41598-021-00593-z (PMC8551274; doi:10.1038/s41598-021-00593-z)
Supplement: Supplementary file 1 — Supplementary Figures. [file 41598_2021_593_MOESM1_ESM.pdf]

Supplementary Figures

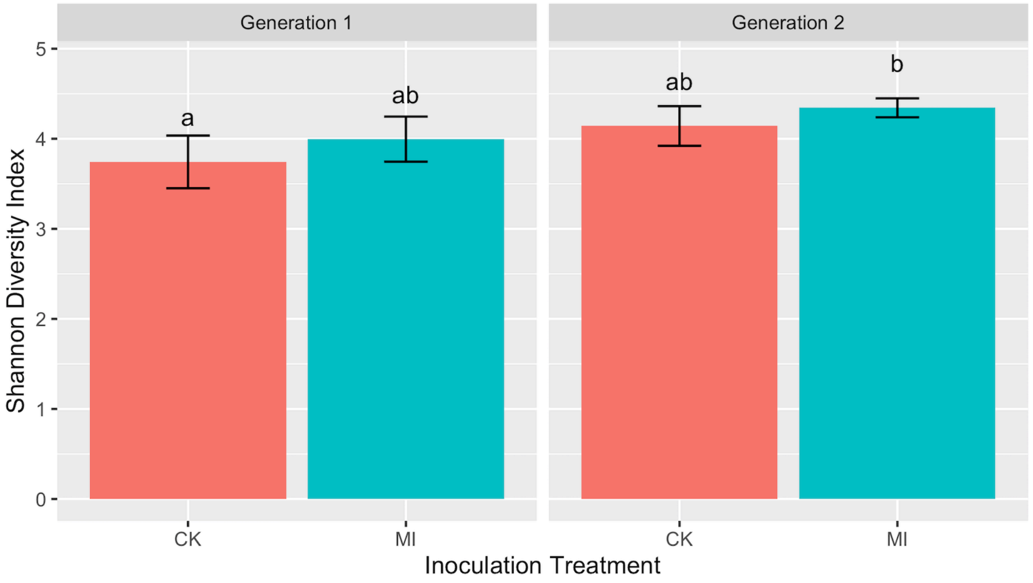

Figure S1. Mean alpha diversity values of each treatment group represented using Shannon Diversity Index values. Values for control (CK) and microbial inoculation (MI) treatments are presented here within generation 1 and generation 2. Different letters indicate significant differences at $p < 0.05$.

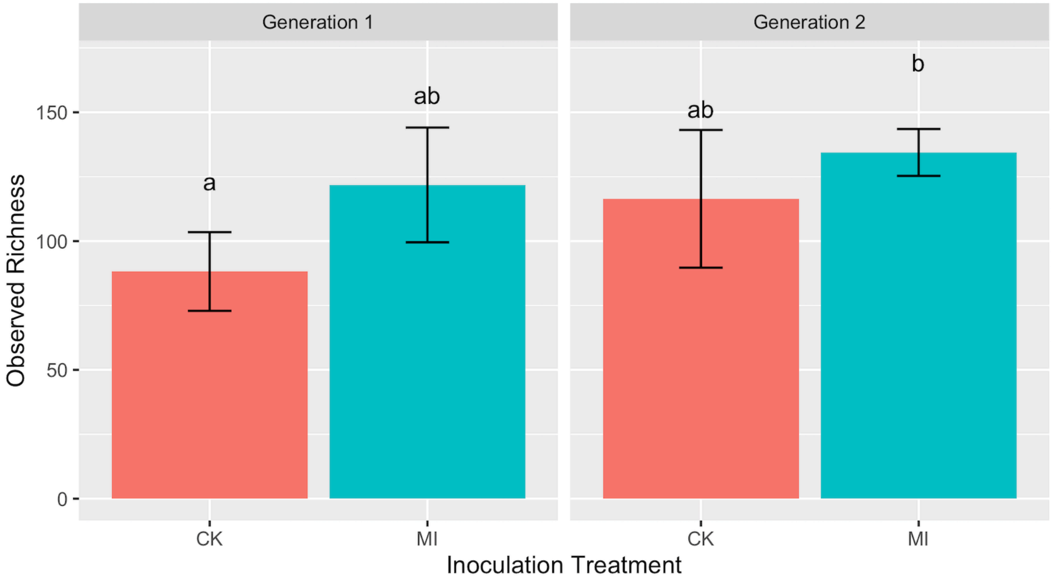

Figure S2. Mean alpha diversity values of each treatment group represented using observed richness values. Values for control (CK) and microbial inoculation (MI) treatments are presented here within generation 1 and generation 2. Different letters indicate significant differences at $p < 0.05$.

a. Generation 1

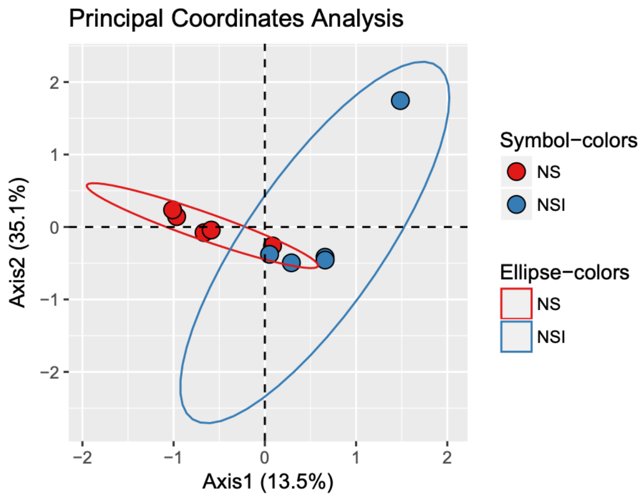

b. Generation 2

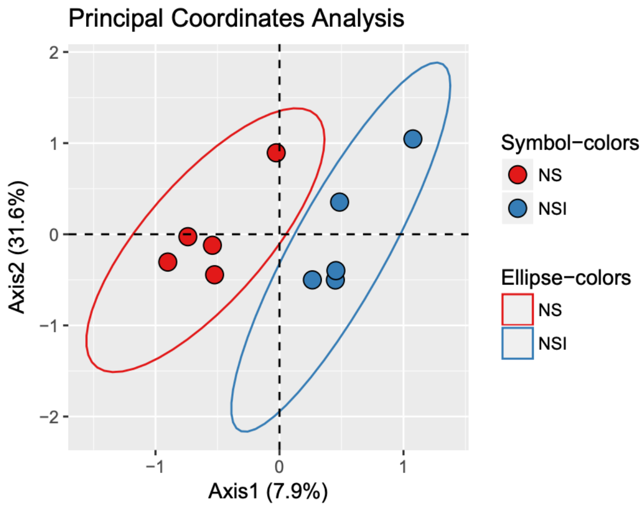

Figure S3. Principal Coordinates Analysis (PCoA), using Bray-Curtis distances, representing rhizobacterial communities of soil samples from inoculated and control treatments (n=5 soil

samples per water treatment) in not autoclaved soils within generation 1 (**a**) and generation 2 (**b**). Red and blue circles represent control (NS) and microbial inoculation (NSI) treatments, respectively.
