# Supplementary material for: Conditioned soils reveal plant-selected microbial communities that impact plant drought response
Source: Sci Rep. 2021 Oct 27;11:21153. doi: 10.1038/s41598-021-00593-z (PMC8551274; doi:10.1038/s41598-021-00593-z)
Supplement: Supplementary file 2 — Supplementary Tables. [file 41598_2021_593_MOESM2_ESM.pdf]

Supplementary Tables

Family_Genus	baseMeanMI	baseMeanCK	logFC	p-value	FDR
Xanthomonadaceae_unclassified	167.04	0	-10.385	8.51E-15	1.8552E-12
Micromonosporaceae_Couchioplanes	99.106	0	-9.633	1.0161E-08	1.1076E-06
Comamonadaceae_Hydrogenophaga	45.792	0	-8.521	2.7084E-07	1.9681E-05
Pirellulaceae_unclassified	17.244	0	-7.119	2.1227E-06	0.00011569
(o: Ellin6067) unclassified_unclassified	14.924	0	-6.912	6.0419E-06	0.00022852
Bryobacteraceae_unclassified	14.59	0	-6.88	6.2896E-06	0.00022852
(o: Gemmatimonadales) unclassified_unclassified	0	31.716	7.992	0.00024082	0.00749997
RB40_unclassified	13.542	0	-6.774	0.00029473	0.00803149
Nocardioidaceae_Aeromicrobium	0	27.51	7.793	0.00039061	0.00946153
Planctomycetaceae_Planctomyces	9.946	0	-6.332	0.00068627	0.01496074
(o: Pedosphaerales) unclassified_unclassified	0	8.19	6.057	0.00099051	0.01963013
Chthoniobacteraceae_unclassified	0	6.87	5.805	0.00213901	0.03885859
Cytophagaceae_unclassified	12.76	0.738	-3.901	0.0030617	0.05134229

Table S1. Bacterial taxa, to the genus level, observed to significantly differ in abundance in the microbial inoculation treatment (MI) as compared to the control (CK) in generation 1 (FDR<0.01; p<0.01).

Family_Genus	baseMeanMI	baseMeanCK	logFC	p-value	FDR
Xanthomonadaceae_unclassified	38.838	0	-8.284	6.3187E-12	1.3775E-09
Rhizobiaceae_Sinorhizobium	0	39.192	8.297	6.7073E-10	7.3109E-08
Rhodospirillaceae_unclassified	26.644	0	-7.742	2.296E-08	1.6384E-06
Methylobacteriaceae_Methylobacterium	0	27.088	7.766	3.0062E-08	1.6384E-06
RB40_unclassified	26.946	0.284	-6.048	3.7389E-07	1.6301E-05
Phyllobacteriaceae_Chelativorans	17.462	0	-7.136	9.5151E-07	3.4571E-05
Pirellulaceae_unclassified	13.768	0	-6.796	3.5576E-06	8.6174E-05
(o: Ellin6067)unclassified_unclassified	13.482	0	-6.766	3.5248E-06	8.6174E-05
Planctomycetaceae_Planctomyces	13.986	0	-6.818	2.9406E-06	8.6174E-05
Chitinophagaceae_unclassified	21.1	0.472	-5.152	5.676E-05	0.00123736
Sphingomonadaceae_Sphingomonas	0	19.238	7.275	9.9778E-05	0.00197742
Sporichthyaceae_unclassified	44.904	2.108	-4.334	0.00021064	0.00382667
Chitinophagaceae_Flavihumibacter	6.384	0	-5.702	0.00023115	0.00387628

Cytophagaceae_unclassified	7.058	0	-5.845	0.00029843	0.00464705
mb2424_unclassified	9.336	0	-6.241	0.00035658	0.00518234
Chthoniobacteraceae_unclassified	0	11.678	6.56	0.00043213	0.00588781
Chitinophagaceae_Flavisolibacter	0.44	15.78	4.816	0.00061873	0.00793433
Cytophagaceae_Larkinella	0	8.496	6.11	0.000835	0.0101128
Bryobacteraceae_unclassified	4.524	0	-5.217	0.00267812	0.03072795
Nocardioidaceae_unclassified	31.312	208.838	2.734	0.00369072	0.03831314
Phyllobacteriaceae_Aminobacter	74.032	5.394	-3.745	0.00354015	0.03831314
Caulobacteraceae_Mycoplana	0	6.874	5.808	0.00418256	0.04144537
(o: Myxococcales) unclassified_unclassified	21.134	1.27	-3.929	0.00608603	0.05768496
Hyphomicrobiaceae_Hyphomicrobium	5.444	0	-5.479	0.00657123	0.05968867
Oxalobacteraceae_Janthinobacterium	9.25	0	-6.231	0.00859442	0.07494332

Table S2. Bacterial taxa, to the genus level, observed to significantly differ in abundance in the microbial inoculation treatment (MI) as compared to the control (CK) in generation 2 (FDR<0.01; p<0.01).

**Family_Genus**	**baseMeanG2**	**baseMeanG1**	**logFC**	**p-value**	**FDR**
Micromonosporaceae_Couchioplanes	0	99.106	9.634	4.3348E-10	9.4498E-08
Nocardioidaceae_Aeromicrobium	19.91	0	-7.324	1.3315E-07	1.0553E-05
Phyllobacteriaceae_Chelativorans	17.462	0	-7.135	1.4522E-07	1.0553E-05
Phyllobacteriaceae_Aminobacter	74.032	11.22	-2.706	1.6929E-06	9.2263E-05
Bradyrhizobiaceae_Balneimonas	1.5	43.894	4.764	1.992E-05	0.0008685
Xanthomonadaceae_unclassified	38.838	167.04	2.102	0.00051865	0.01884435
Oxalobacteraceae_unclassified	27.08	219.738	3.017	0.00105321	0.03280004
Nocardioidaceae_unclassified	31.312	222.544	2.827	0.0015197	0.04141192

Table S3. Bacterial taxa, to the genus level, observed to significantly differ in abundance in inoculated soils from generation 1 (G1) compared to inoculated soils from generation 2 (G2) (FDR<0.01; p<0.01).

**Family_Genus**	**baseMeanG1**	**baseMeanGF**	**logFC**	**p-value**	**FDR**
Paenibacillaceae_Ammoniphilus	10.118	0	-6.359	4.2167E-06	6.7994E-05
Planococcaceae_Planomicrobium	5.93	0	-5.602	0.00798295	0.03739065

Micrococcaceae_Arthrobacter	5.832	0	-5.579	0.00887632	0.04017705
Rhizobiaceae_Sinorhizobium	5.15	0	-5.403	0.01155735	0.04828981
Oxalobacteraceae_Janthinobacterium	5.064	0	-5.377	0.00376108	0.01993541
unclassified_unclassified	50.598	1.18	-5.284	6.7833E-14	8.7505E-12
Rhodobacteraceae_Rhodobacter	4.426	0	-5.188	0.00499053	0.02504958
Oxalobacteraceae_unclassified	218.99	7.746	-4.804	5.4049E-08	1.3945E-06
Bacillaceae_unclassified	4.928	0.066	-4.727	0.00912833	0.04060531
unclassified_unclassified	2.004	0	-4.093	0.0034506	0.01894159
Brucellaceae_Ochrobactrum	1.922	0	-4.037	0.02279772	0.08044487
Bradyrhizobiaceae_Balneimonas	44.14	2.604	-4.025	3.1595E-07	6.1966E-06
unclassified_unclassified	1.582	0	-3.774	0.02295371	0.08044487
Planococcaceae_unclassified	1.37	0	-3.583	0.02780158	0.09471523
Alteromonadaceae_Cellvibrio	8.506	0.686	-3.416	0.00489434	0.02504958
Cytophagaceae_unclassified	12.818	1.3	-3.188	3.9033E-05	0.00050352
Cytophagaceae_Adhaeribacter	4.252	0.42	-3.01	0.0081158	0.03739065
Pseudomonadaceae_Pseudomonas	16.546	2.254	-2.812	0.00327871	0.0183893
Bacillaceae_Bacillus	139.366	21.884	-2.668	3.3625E-07	6.1966E-06
Bradyrhizobiaceae_unclassified	9.62	1.872	-2.29	0.01160453	0.04828981
Micrococcaceae_unclassified	198.59	43.602	-2.188	5.6076E-05	0.00068893
Nocardioidaceae_Pimelobacter	45.61	11.312	-2.004	0.012386	0.05072361
Pirellulaceae_unclassified	18.06	5.284	-1.753	0.00285038	0.01691943
Sphingomonadaceae_unclassified	6.036	21.112	1.781	0.01766111	0.06603718
Comamonadaceae_Ramlibacter	6.146	23.124	1.886	0.00378618	0.01993541
Comamonadaceae_Hydrogenophaga	45.164	182.798	2.01	0.01365797	0.05505869
Sphingomonadaceae_Sphingobium	8.65	38.228	2.124	1.6493E-05	0.0002364
Erythrobacteraceae_unclassified	2.936	16.278	2.418	0.00642379	0.03127054
Sphingomonadaceae_Sphingopyxis	8.574	47.674	2.454	0.00010366	0.00102862
Verrucomicrobiaceae_unclassified	1.526	10.182	2.639	0.01714454	0.065108
Flavobacteriaceae_Flavobacterium	0	0.762	2.825	0.02790061	0.09471523
Sphingomonadaceae_Novosphingobium	0	0.822	2.92	0.02307334	0.08044487
Ellin517_unclassified	0	0.854	2.968	0.02090783	0.07597494
Geodermatophilaceae_unclassified	0	0.868	2.988	0.02003558	0.07384541
Beijerinckiaceae_unclassified	0	0.92	3.062	0.01716025	0.065108
Rhizobiaceae_Kaistia	0	0.936	3.084	0.01637532	0.0640126
Rhizobiaceae_Agrobacterium	2.524	22.58	3.096	7.1199E-05	0.00083497
unclassified_unclassified	6.146	54.322	3.112	0.00667348	0.03188441
Caulobacteraceae_Arthrospira	0	0.994	3.161	0.0138753	0.05507428
Xanthobacteraceae_Ancylobacter	0	1.012	3.183	0.01078843	0.04639026
Pseudonocardiaceae_Pseudonocardia	0	1.042	3.221	0.01070389	0.04639026
C111_unclassified	0	1.366	3.574	0.00315497	0.01808847
Sinobacteraceae_unclassified	0	1.41	3.616	0.00288548	0.01691943
unclassified_unclassified	1.24	16.688	3.618	3.0394E-05	0.00041272
Microbacteriaceae_Agrococcus	0	1.494	3.693	0.00504875	0.02504958

Thermoactinomycetaceae_unclassified	0	1.69	3.858	0.00121966	0.00806853
unclassified_unclassified	0	1.718	3.88	0.00108576	0.00737171
Cellulomonadaceae_Actinotalea	0	1.728	3.888	0.00177654	0.01091303
unclassified_unclassified	0	1.844	3.976	0.00079821	0.00588396
Verrucomicrobiaceae_Luteolibacter	0	1.882	4.003	0.00077314	0.00586678
Hyphomicrobiaceae_Rhodoplanes	0	1.98	4.072	0.00137587	0.00865789
Comamonadaceae_Methylibium	0	2.318	4.287	0.00033635	0.00271182
Bradyrhizobiaceae_Bosea	0	2.322	4.289	0.00044528	0.00348128
unclassified_unclassified	0	2.336	4.297	0.00127273	0.00820911
Microbacteriaceae_Microbacterium	0	2.558	4.422	0.00082456	0.00590938
unclassified_unclassified	0	2.774	4.534	0.00018618	0.00165636
Verrucomicrobiaceae_Prosthecobacter	0	2.924	4.606	0.00106072	0.00737171
Rhizobiaceae_Rhizobium	0	3.006	4.645	0.00024112	0.00207359
Cytophagaceae_Dyadobacter	1.038	31.538	4.763	1.4175E-08	4.5715E-07
Phyllobacteriaceae_Chelativorans	0	3.294	4.772	8.5425E-05	0.00088158
Comamonadaceae_Variovorax	0	3.454	4.838	0.0002938	0.00244515
Methylophilaceae_Methylotenera	0	3.484	4.85	8.2896E-05	0.00088158
Rhizobiaceae_Shinella	0	3.726	4.943	8.2392E-05	0.00088158
Nocardioidaceae_Aeromicrobium	0	4.336	5.156	0.00017539	0.00161613
Rhodobacteraceae_Rubellimicrobium	0	5.266	5.429	9.4998E-06	0.00014417
Streptomycetaceae_Streptomyces	0	5.498	5.489	0.00012636	0.0012074
Comamonadaceae_Delftia	0	5.722	5.546	3.0901E-06	5.3149E-05
Sphingobacteriaceae_unclassified	0	7.822	5.989	9.5248E-08	2.0478E-06
unclassified_unclassified	0	8.036	6.027	6.4036E-08	1.5019E-06
Caulobacteraceae_Mycoplana	0	11.266	6.508	1.7315E-08	4.9637E-07
Comamonadaceae_Pelomonas	0	12.126	6.613	7.6203E-09	2.8086E-07
Sphingomonadaceae_Sphingomonas	0	16.644	7.066	2.9947E-12	1.5453E-10
Caulobacteraceae_unclassified	0	21.32	7.421	1.9239E-14	4.9638E-12
Nocardioidaceae_Nocardioides	0	23.12	7.537	4.572E-09	1.966E-07
unclassified_unclassified	0	24.022	7.592	1.0712E-12	6.9091E-11
Pseudonocardiaceae_Amycolatopsis	0	27.154	7.768	1.153E-13	9.9156E-12

Table S4. Bacterial taxa, to the genus level, observed to significantly differ in abundance in inoculated soils from the generational foundation soil slurry (GF) compared to inoculated soils from generation 1 (G1) (FDR<0.01; p<0.01).

**Family_Genus**	**baseMeanG2**	**baseMeanGF**	**logFC**	**p-value**	**FDR**
AK1AB1_02E_unclassified	0.636	0	-2.605	0.03601489	0.09679
Alteromonadaceae_Cellvibrio	0.686	12.31	3.94	4.815E-05	0.00035493

Bacillaceae_Bacillus	21.884	240.288	3.451	4.9658E-27	6.4059E-25
Bacillaceae_unclassified	0.066	16.08	6.408	4.3104E-11	7.489E-10
Beijerinckiaceae_unclassified	0.92	0	-3.063	0.01198924	0.03818795
Bradyrhizobiaceae_Bosea	2.322	0	-4.29	0.00020808	0.00116705
Bradyrhizobiaceae_unclassified	1.872	10.324	2.389	0.02662663	0.0730816
Bryobacteraceae_unclassified	25.526	4.604	-2.438	6.2277E-06	6.427E-05
Caulobacteraceae_Arthrospira	0.994	0	-3.161	0.00912347	0.03017762
Caulobacteraceae_Mycoplana	11.266	0	-6.509	2.1006E-09	3.0109E-08
Caulobacteraceae_Phenylobacterium	14.686	2.844	-2.317	0.00545352	0.01876009
Caulobacteraceae_unclassified	21.32	0	-7.422	1.5395E-15	5.6743E-14
Cellulomonadaceae_Actinotalea	1.728	0	-3.889	0.00121097	0.00529544
Chitinophagaceae_Flavisolibacter	3.098	0.414	-2.578	0.00497629	0.01758744
Chitinophagaceae_unclassified	3.442	21.188	2.58	6.1202E-06	6.427E-05
Comamonadaceae_Azohydromonas	1.612	0	-3.796	0.00126196	0.00542642
Comamonadaceae_Delftia	5.722	0	-5.547	1.3004E-06	1.4587E-05
Comamonadaceae_Hydrogenophaga	182.798	84.964	-1.102	0.00024058	0.00129309
Comamonadaceae_Methylibium	2.318	0	-4.288	0.00017014	0.00099766
Comamonadaceae_Pelomonas	12.126	0	-6.614	9.1924E-10	1.4823E-08
Comamonadaceae_Variovorax	3.454	0	-4.839	0.0001147	0.00070461
Cytophagaceae_Adhaeribacter	0.42	3.232	2.623	0.02513073	0.0697175
Cytophagaceae_Dyadobacter	31.538	0.23	-6.477	4.5773E-19	2.9524E-17
Cytophagaceae_unclassified	1.3	6.906	2.305	0.00472882	0.01694494
Flavobacteriaceae_Flavobacterium	0.762	0	-2.826	0.02188918	0.06274899
Gemmatimonadaceae_Gemmatimonas	15.204	36.184	1.246	0.00051547	0.00247089
Geodermatophilaceae_unclassified	0.868	0	-2.989	0.0145757	0.04372711
Haliangiaceae_unclassified	4.846	0	-5.313	8.6114E-06	7.9348E-05
Hyphomicrobiaceae_Rhodoplanes	1.98	0	-4.073	0.00050772	0.00247089
Hyphomonadaceae_unclassified	1.632	10.422	2.588	7.7205E-05	0.00051074
mb2424_unclassified	1.52	9.534	2.555	0.00185665	0.00760342
Methylobacteriaceae_unclassified	3.944	0	-5.024	2.4408E-05	0.00019679
Methylophilaceae_Methylotenera	3.484	0	-4.851	2.3013E-05	0.00019152
Microbacteriaceae_Agrococcus	1.494	0	-3.694	0.00270934	0.01059107
Microbacteriaceae_Microbacterium	2.558	0	-4.423	0.00026218	0.00138047
Microbacteriaceae_unclassified	0	6.748	5.783	0.00140776	0.00595414
Micrococcaceae_Arthrobacter	0	2.002	4.09	0.01355703	0.04165031
Micrococcaceae_unclassified	43.602	186.794	2.097	6.9996E-05	0.00048808
Micromonosporaceae_Couchioplanes	65.412	0	-9.033	8.0941E-32	2.0883E-29
Micromonosporaceae_unclassified	572.368	197.006	-1.536	4.8223E-09	6.5482E-08
Nocardioidaceae_Aeromicrobium	4.336	20.374	2.202	0.00052674	0.00247089
Nocardioidaceae_Nocardioides	23.12	0	-7.538	1.8973E-09	2.8795E-08
Nocardioidaceae_Pimelobacter	11.312	37.204	1.708	0.00019152	0.00109805
Nocardioidaceae_unclassified	130.06	30.962	-2.065	0.00301883	0.01162475
Opitutaceae_Opitutus	9.902	27.936	1.486	0.00049379	0.00247089
Oxalobacteraceae_Janthinobacterium	0	8.956	6.186	0.00091614	0.00414675
Oxalobacteraceae_unclassified	7.746	27.594	1.819	0.00436972	0.01587869
Paenibacillaceae_Ammoniphilus	0	29.85	7.906	2.0237E-19	1.7404E-17

Paenibacillaceae_Aneurinibacillus	0	3.438	4.833	0.00525311	0.01831489
Paenibacillaceae_Cohnella	2.91	15.06	2.323	0.02229619	0.06321336
Phyllobacteriaceae_Aminobacter	10.326	75.094	2.849	1.257E-12	2.7026E-11
Phyllobacteriaceae_Chelativorans	3.294	17.53	2.37	1.2099E-05	0.00010764
Phyllobacteriaceae_Mesorhizobium	4.618	0	-5.245	8.0234E-06	7.6668E-05
Phyllobacteriaceae_unclassified	1.268	0	-3.477	0.00375923	0.01405624
Pirellulaceae_unclassified	5.284	13.888	1.375	0.00931054	0.03040656
Planctomycetaceae_Planctomyces	4.388	14.2	1.668	0.0040647	0.01498132
Planococcaceae_unclassified	0	6.612	5.752	0.00021984	0.00120679
Pseudomonadaceae_Pseudomonas	2.254	23.584	3.32	0.00016972	0.00099766
Pseudonocardiaceae_Amycolatopsis	27.154	0	-7.769	1.8338E-15	5.9139E-14
Pseudonocardiaceae_Pseudonocardia	1.042	0	-3.222	0.00815265	0.02767611
RB40_unclassified	5.482	26.708	2.261	1.8216E-07	2.3498E-06
Rhizobiaceae_Agrobacterium	22.58	0	-7.504	5.6198E-17	2.4165E-15
Rhizobiaceae_Kaistia	0.936	0	-3.085	0.0112961	0.03642992
Rhizobiaceae_Rhizobium	3.006	0	-4.646	8.8435E-05	0.00057041
Rhizobiaceae_Shinella	3.726	0	-4.944	2.1151E-05	0.0001819
Rhodobacteraceae_Rhodobacter	0	19.46	7.293	1.169E-13	3.016E-12
Rhodobacteraceae_Rubellimicrobium	5.266	0	-5.43	6.6461E-06	6.595E-05
Sinobacteraceae_unclassified	1.41	0	-3.617	0.00233144	0.00939862
Sphingobacteriaceae_Pedobacter	1.558	0	-3.75	0.00171051	0.00711793
Sphingomonadaceae_Novosphingobium	0.822	0	-2.921	0.01736482	0.05091049
Sphingomonadaceae_Sphingobium	38.228	19.688	-0.951	0.01356057	0.04165031
Sphingomonadaceae_Sphingomonas	16.644	0	-7.067	3.0398E-13	7.1298E-12
Sphingomonadaceae_Sphingopyxis	47.674	3.924	-3.559	4.354E-11	7.489E-10
Sphingomonadaceae_unclassified	21.112	6.87	-1.6	0.01592416	0.04722338
Sporichthyaceae_unclassified	23.95	45.032	0.909	0.03014229	0.08186012
Staphylococcaceae_Staphylococcus	2.296	32.736	3.764	7.0781E-12	1.4047E-10
Streptomycetaceae_Streptomyces	5.498	0	-5.491	0.00010882	0.00068476
Thermoactinomycetaceae_unclassified	1.69	0	-3.859	0.0010567	0.00470047
unclassified_unclassified	1.18	39.214	4.916	6.114E-18	3.1548E-16
unclassified_unclassified	24.022	0	-7.593	1.7616E-14	5.0499E-13
unclassified_unclassified	54.322	4.188	-3.657	2.4553E-07	3.0166E-06
unclassified_unclassified	49.7	103.926	1.064	3.1818E-05	0.00024876
unclassified_unclassified	2.774	0	-4.535	4.7517E-05	0.00035493
unclassified_unclassified	0	4.762	5.29	7.6005E-05	0.00051074
unclassified_unclassified	1.844	0	-3.977	0.00062217	0.00286642
unclassified_unclassified	347.132	577.328	0.736	0.0025711	0.01020528
unclassified_unclassified	16.688	5.446	-1.592	0.0032777	0.01243599
unclassified_unclassified	0	1.828	3.967	0.01320041	0.041533
Verrucomicrobiaceae_Luteolibacter	1.882	0	-4.004	0.00052025	0.00247089
Verrucomicrobiaceae_Prosthecobacter	2.924	0	-4.607	0.00035023	0.00180719
Verrucomicrobiaceae_unclassified	10.182	1.578	-2.595	0.01374791	0.04172896
Xanthobacteraceae_Ancylobacter	1.012	0	-3.184	0.00905233	0.03017762
Xanthomonadaceae_Dokdonella	0.194	3.858	3.645	0.02116284	0.06134847
Xanthomonadaceae_Lysobacter	24.184	7.4	-1.689	0.02494179	0.0697175

Xanthomonadaceae_Pseudoxanthomonas	25.962	0.728	-4.933	1.0901E-06	1.2784E-05
Xanthomonadaceae_unclassified	93.162	39.568	-1.231	5.1424E-05	0.00036854

Table S5. Bacterial taxa, to the genus level, observed to significantly differ in abundance in inoculated soils from the generational foundation soil slurry (GF) compared to inoculated soils from generation 2 (G2) (FDR<0.01; p<0.01).

**Family_Genus**	**baseMeanGF**	**baseMeanNA_G2**	**logFC**	**p-value**	**FDR**
Phyllobacteriaceae_Chelativorans	0.364	0	-1.968	1.12E-01	5.22E-01
Phyllobacteriaceae_Aminobacter	1.086	0.482	-0.997	2.80E-01	1
**Family_Genus**	**baseMeanNA_G2**	**baseMeanNA_G1**	**logFC**	**p-value**	**FDR**
Phyllobacteriaceae_Chelativorans	0	0	0	1	1
Phyllobacteriaceae_Aminobacter	0.482	0	-2.279	0.124986	1
**Family_Genus**	**baseMeanGF**	**baseMeanNA_G1**	**logFC**	**p-value**	**FDR**
Phyllobacteriaceae_Chelativorans	0.364	0	-1.967	1.14E-01	6.67E-01
Phyllobacteriaceae_Aminobacter	1.086	0	-3.275	6.12E-03	8.26E-02

Table S6. Differences in abundance for *Phyllobacteriaceae_Chelativorans* and *Phyllobacteriaceae_Aminobacter* in inoculated treatment groups from each generation in not autoclaved soils: initial soil slurry (GF), generation 1 (NA_G1) and generation 2 (NA_G2).

**Family_Genus**	**baseMeanMI**	**baseMeanCK**	**logFC**	**p-value**	**FDR**
Phyllobacteriaceae_Chelativorans	0	0	0	1	1
Phyllobacteriaceae_Aminobacter	4.508	0	-5.212	0.18269	1

Table S7. Differences in abundance for *Phyllobacteriaceae_Chelativorans* and *Phyllobacteriaceae_Aminobacter* in microbial inoculation (MI) and control (CK) treatment groups in generation 2 for not autoclaved soils.
